# As Women Live Longer, What Do They Need Most?

**DOI:** 10.1093/geroni/igab046.3666

**Published:** 2021-12-17

**Authors:** Jennifer Yahner, Jeanette Hussemann

**Affiliations:** 1 Urban Institute, Washington, District of Columbia, United States; 2 NORC at the University of Chicago, Chicago, Illinois, United States

## Abstract

Women in the United States can live into their 80s, 90s, and even 100s—outliving men nearly five years on average. Over the next four decades, the number of women aged 85 years and older will nearly triple in size. Many will live alone and in poverty, with increasingly fewer supports on which to rely as they age. Although women can spend their lives caring for children, partners, and parents, often while working multiple jobs, as they grow older, many find their physical, emotional, and financial needs cannot be met. Using data recently collected for the Urban Institute’s EMPOWER: Building Late-Life Resilience study, with funding from the National Institute of Justice, we examine the needs of low-income women aged 85 years and older (N=35) living alone in Arizona communities. We explore issues of home safety perceptions and social isolation and study their relationship to women’s physical, emotional, and financial wellbeing.

